# Supplementary material for: Performance evaluation of nine reference centers and comparison of DNA extraction protocols for effective surveillance of Leishmania-infected Phlebotomine sand flies: Basis for technical recommendations
Source: PLoS Negl Trop Dis. 2024 Dec 23;18(12):e0012543. doi: 10.1371/journal.pntd.0012543 (PMC11706373; doi:10.1371/journal.pntd.0012543)
Supplement: Attachment — Submitted filename: Prudhomme et al review Plos NTD 2024.docx [file pntd.0012543.s002.docx]

**Review of: Performance evaluation of nine reference centers for effective surveillance of**

***Leishmania*-infected Phlebotomine sand flies and basis for technical**

**Recommendations by Prudhomme et al.**

Minor Revisions:

Line 41, 134, 140: Leishmania infantum and major are different species, not strains. Please replace.

Line 49, ‘sand fly’ should be ‘sand flies’

Line 61, infected is misspelled.

Line 144, shoud read “Ready to use,”

Line 153 and 194, grinded should instead by ‘ground’

Table 2, title or legend should indicate what is being compared (Cq value) in the table.

Table 2, to make it easier to read and find the comparisons of interest you could split the Kit Compared column into two, and merge the cells for each of kit 1. Example:

| EZ1 DSP Virus® Kit | EZ1 DSP Virus® Kit |
| --- | --- |
|  | EZ1 DSP Virus® Kit |
|  | EZ1 DSP Virus® Kit |
|  | EZ1 DSP Virus® Kit |
|  | EZ1 DSP Virus® Kit |
|  | EZ1 DSP Virus® Kit |
| RSC Blood DNA® v | EZ1 DSP Virus® Kit |
|  | EZ1 DSP Virus® Kit |
|  | EZ1 DSP Virus® Kit |
|  | EZ1 DSP Virus® Kit |
|  | EZ1 DSP Virus® Kit |
|  | EZ1 DSP Virus® Kit |
| QIAamp® viral RNA mini kit v | EZ1 DSP Virus® Kit |
|  | EZ1 DSP Virus® Kit |
|  | EZ1 DSP Virus® Kit |
|  | EZ1 DSP Virus® Kit |
|  | EZ1 DSP Virus® Kit |
|  | EZ1 DSP Virus® Kit |

Line 241, should read in the presence of “sand fly DNA”

Line 258, should read “such as inaccurate”

Major Revisions:

Did any centers have amplification/obtain a Cq value on the DNA from the uninfected sand fly DNA? In my experience, unfed or male sand flies sometimes amplify with a late Cq value using kDNA primers. Reporting the background amplification Cq obtained on the negative flies with the different extraction kits could be valuable information and determine if the higher Cq obtained by Center 1 in the Fig 4 is actually above background. It could be added in the same graph. If no amplification of negative sand fly DNA was observed by any centers, that is also worth mentioning in the results section.
