# Supplementary material for: Performance evaluation of nine reference centers and comparison of DNA extraction protocols for effective surveillance of Leishmania-infected Phlebotomine sand flies: Basis for technical recommendations
Source: PLoS Negl Trop Dis. 2024 Dec 23;18(12):e0012543. doi: 10.1371/journal.pntd.0012543 (PMC11706373; doi:10.1371/journal.pntd.0012543)
Supplement: Attachment — Submitted filename: Reponse to reviewers.docx [file pntd.0012543.s003.docx]

23 October 2024

**Object:** Answer to reviewer for manuscript PNTD-D-24-01313

Dear Editor,

We thank the reviewers for their comments on our manuscript no. PNTD-D-24-01313 “Performance evaluation of nine reference centers for effective surveillance of *Leishmania*-infected Phlebotomine sand flies and basis for technical recommendations”. We have tried to answer to their comments as fully as possible. In the following section you will find our responses to each point raised and references to the changes we have made in the manuscript.

Best regards,

On behalf of co-authors

Jorian Prudhomme

Dr. Prudhomme Jorian,

Tel: +33(0)621504351

[jorian.prudhomme@hotmail.fr](mailto:jorian.prudhomme@hotmail.fr)

**Reviewer #1:**

**==============**

**In their paper entitled "Performance evaluation of nine reference centres for effective surveillance of Leishmania-infected Phlebotomine sand flies and basis for technical recommendations", Prudhomme & Delabarre et al. present the findings of a multicentre study involving nine reference laboratories. The aim of the study was twofold: firstly, to compare various DNA extraction protocols and, secondly, to conduct a qPCR-based external quality assessment.**

**The manuscript is of great interest and is well written, although it would benefit from a few minor revisions as suggested.**

**Lane 156: it would be useful to include the names of the participating centres in the table.**

Answer: The name of the participating centers were added in Table 1.

**Lane 157: the PCR protocol, which has been previously described by Mary et al. (2004), is a highly sensitive method. However, no information regarding its specificity is provided in the initial article or in this study. It would be valuable to understand how the authors excluded aspecific amplification and whether they sequenced random samples.**

Answer: This method has now a long history of employment for human diagnosis and is widely used across Europe and beyond without any problem of specificity, as reported in many studies (De Pascali AM, et al. PLoS NTD 2022; Gow I, et al. Parasit Vectors 2022; Melkamu R, et al. PLoS NTD 2023; Tanida K, et al. Pathogens 2021). In the last paper, it was negative in 1137 subjects with no *Leishmania* infection. We also use this qPCR in our lab (which is a reference lab for leishmaniasis diagnosis in an academic hospital) since a long time and have previously proven its sensitivity and specificity for accreditation. We thus believe that sequencing in the context of the current study is not pertinent, all the more that we used *Leishmania*-spiked samples.

**With regard to the comparison of extraction protocols (Lane 219), it would be helpful to know whether the authors assessed the purity and concentration of the DNA obtained through 260/280 nm absorbance measures using a NanoDrop spectrophotometer. This value should be taken into consideration when comparing the performance of the different extraction protocols. I suggest providing further detail on this and to discuss the concerning results in greater depth.**

Answer: We thank the reviewer for his valuable feedback. While we appreciate the suggestion to assess DNA purity and concentration through 260/280 nm absorbance measurements using a NanoDrop spectrophotometer, we believe that this specific evaluation is not directly relevant to our study's primary objectives. Our focus was to compare the efficiency of different DNA extraction protocols and their effectiveness in detecting *Leishmania* DNA in sand flies through qPCR, as indicated by the quantification cycle (Cq) values. The Cq values provide a direct measure of the sensitivity and robustness of the DNA detection methods used in this study, which is our main concern when evaluating the performance of the different extraction protocols. While purity and concentration are certainly important factors in molecular biology, our primary goal was to determine the operational efficiency and compatibility of various extraction methods in the context of *Leishmania* DNA detection. Since all participating laboratories adhered to standardized protocols for qPCR, the relative differences in Cq values directly reflect the comparative performance of the extraction kits, regardless of the initial purity and concentration of the DNA. Therefore, we believe that including absorbance measurements may not significantly enhance the findings presented in our study, all the more that some parasite concentrations were low (10/mL, 100/mL).

**Reviewer #2:**

**==============**

**Leishmaniasis is a neglected diseases that requires more in-depth research in all directions including diagnosis. This is what make the study important and interesting. However, several comments below should be considered:**

**Abstract**

**Line 35: Would it be good to mention the other common name for qPCR, real time PCR?**

Answer: We changed the sentence “Consistent quantitative real-time PCR…”

**Line 36: replace the word ‘condition’ by a word related to method of diagnosis or detection.**

Answer: The word “condition” was replaced by “parameters”.

**Line 38-39: In the aim, would better to insert 'DNA in sandflies' somewhere. The title of the manuscript (evaluation of nine centers) and the aim (compare various DNA extraction protocols) should run parallel. It would be helpful to modify one of them to match the other.**

Answer: We inserted “*Leishmania* DNA in sand flies” in the abstract.

Answer: The title was changed, thanks to the suggestion of the reviewer, as: “Performance evaluation of nine reference centers and comparison of DNA extraction protocols for effective surveillance of *Leishmania*-infected phlebotomine sand flies: basis for technical recommendations”.

**The abstract does not mention sandflies!**

Answer: As suggested, we inserted “*Leishmania* DNA in sand flies” in the abstract.

**The conclusion and significance are general wording that do not convey a real scientific message.**

Answer: We thank the reviewer for his comment. The abstract was rewritten to convey a real scientific message.

**Author summary**

**Line 61: Correct 'nfected' to 'infected'**

Answer: The word “infected” had been corrected.

**Line 71: add " and proper treatment'.**

Answer: As requested, we changed the sentence “Continuous training and calibration are essential to ensure uniform diagnostic performance across laboratories, enhancing epidemiological surveillance and disease control strategies and enabling appropriate treatment.”

**Introduction:**

**The introduction should enriched with more studies that compared DNA extraction methods and amplification methods.**

Answer: A sentence and two references have been added on the comparison of extraction methods, and several on amplification methods.

**Line 107: to conduct EQA, but for what?**

Answer: Line X was corrected with reviewer comment “…to conduct an External Quality Assessment (EQA) aimed at evaluating the effectiveness and consistency of *Leishmania* DNA detection methods across different laboratories. This multicenter study involved nine reference laboratories, participating in the European project CLIMOS (http://www.climos-project.eu) which collects data on sand fly infection, and aimed at ensuring the reliability and comparability of *Leishmania* detection methods from sand flies across different regions and laboratories, thereby enhancing the accuracy of epidemiological surveillance and contributing to more effective disease control strategies.”

**Again, the aim in the introduction is different from that in the abstract, one talks about comparing extraction methods while the other about Leishmania detection methods. The title mention evaluation of nince centers. Re-phrase your aim.**

Answer: As requested by the reviewer, the aim of the abstract and the title of the article were corrected to ensure consistency between the aims presented in the introduction and the abstract.

**Methods:**

**Line 118: Is the he Rennes Lab the same as INSERM?**

Answer: For a better understanding, the Rennes lab was changed by INSERM.

**Line 120: Why 'the s--called"? Is a reference laboratory or not?**

Answer: The term so-called was deleted.

**Line 151: PBS is phosphate buffered saline?**

Answer: The precision was added in the sentence “…sand fly in 200 µL of phosphate buffered saline (PBS)”.

**Line 154: Do you mean (200 μL of EQA sample)?**

Answer: We corrected the sentence “200 µL of EQA sample and 200 µL lysis buffer”.

**Table 1: Handmade means manual work/extraction. May be the later expression is better.**

Answer: Handmade was replaced by manual extraction in Table 1.

**Tissue lyser II is not an extraction device but rather a disruptor?**

Answer: Tissue lyser II was replaced by manual extraction as this device is not an extraction device.

**Line 158: It will be an added value to justify why you selected this method in particular knowing that we have many good one out there? Is it the method regularly used by all the participating labs?**

Answer: We selected this method because it has been evaluated (by us and others, *e.g.* Tanida et al. 2021) and is routinely used in our laboratory, which is a reference lab for leishmaniasis diagnosis in humans. Furthermore, this real-time quantitative PCR amplification protocol has been extensively validated for high analytical sensitivity, detecting as low as 0.0125 parasites per mL of blood (Reithinger and Dujardin, 2007). This level of sensitivity is crucial for applications involving low parasite loads, such as in epidemiological surveillance and field studies. Moreover, this qPCR demonstrates excellent linearity, providing consistent and reproducible results across a wide range of parasite concentrations. The ability to detect and quantify parasite DNA accurately over a large dynamic range makes it particularly suited for comparative analyses between different laboratories, which was a key objective in our study. This method targets kinetoplast DNA (kDNA) sequences, which are abundant, specific to the genus *Leishmania*, and highly repetitive. These features make kDNA an ideal target for nucleic acid-based detection (Weirather et al., 2011). The high copy number of kDNA within the parasite's mitochondria increases the likelihood of detection, particularly in samples with low parasite burdens, enhancing both the sensitivity and reliability of the assay. Although several good methods exist, we selected this protocol because it not only meets the high standards required for sensitivity and reproducibility but also aligns with the established practices of many of the participating laboratories. This standardization across labs ensures comparability of results and strengthens the reliability of the multicenter study findings.

**Line 164: Cq is quantification cycle. While, threshold cycle is Ct. Both are the same.**

Answer: The sentence was corrected “The qPCR quantification cycle (Cq)…”

**Line 187: How was the shipment condition monitored? Was there a temperature data logger/indicator? Shipment condition might not be at room temperature, but higher in summer.**

Answer: We thank the reviewer for his questions. While the shipment was not actively monitored, it was handled by a professional carrier. Most samples were received within a few days and transported in insulated packages. Additionally, the samples were freeze-dried, making them less susceptible to temperature variations. Only one shipment took longer than a few days (for IMOH), but this center yielded good results. Anyway, we had anticipated this issue and show that room temperature storage in Rennes (at around 25°C for 3 weeks) did not impact the results significantly (fig 4B).

**Results**

**Line 214: Why is L. major Cq is higher than L. infantum? Could be explained in the discussion section.**

Answer: The observation that the Cq (quantification cycle) values for *Leishmania* *major* are higher than those for *Leishmania* *infantum* can be attributed to several factors. One key aspect involves the minicircle kinetoplast DNA (kDNA), which is commonly targeted in qPCR assays for *Leishmania* detection. The copy number of kDNA can vary significantly between species within the *Leishmania* genus, or even between isolates of the same species. It is possible that *L. infantum* has a higher kDNA copy number compared to *L. major*, resulting in lower Cq values for *L. infantum*. Additionally, the efficiency of the qPCR assay may differ between species due to variations in the target DNA sequences. Although the assay targets conserved regions, slight differences in the kDNA sequences of *L. major* and *L. infantum* could affect the binding efficiency of the primers and probes. The primers and probes used in this study were originally optimized for *L. infantum*, meaning they may bind more efficiently to *L. infantum* DNA, leading to faster amplification and lower Cq values. In contrast, the binding affinity for *L. major* might be lower, resulting in delayed amplification and higher Cq values. This was added in the discussion.

**Line 223: Any explanation why DNA concentration was not determined after the extraction? DNA yield and quality are direct parameters to assess efficiency of DNA extraction method.**

Answer: As stated in our response to Reviewer 1, we thank the reviewer for their valuable feedback. While we appreciate the suggestion to assess DNA concentration, we believe that this specific evaluation is not directly relevant to our study's primary objectives. While purity and concentration are certainly important factors in molecular biology, these parameters are indirectly evaluated through the amplification efficiency, *i.e.* the Cq result. Additionally, some parasite concentrations were very low (10/mL, 100/mL) in a volume of 200 µL, thus the accuracy of DNA dosage using nanodrop would have been questionable and insufficient.

**Tables 2 and 3: can be put as supplements and turn this table into a table or figure that highlights sensitivity. Cq highly variable between centers. Explain.**

Answer: We thank the reviewer for his comment. However, we believe these data are necessary to demonstrate the differences between the various kits used in the study. Additionally, sensitivity by species for each of the centers is highlighted in Figures 1 and 2. As mentioned by the reviewer, Cq values vary between centers. We had already included some elements in the discussion that could explain this observation (lines 274-275, “Lower sensitivity in qPCR amplification detection could be related to lower performance of the DNA extraction method or of the qPCR master mix used” and lines 286-288, “The observed differences in mean Cq values could be attributable to the technical proficiency of the operator, the differences between equipment and positive threshold setting, the DNA extraction kit used, or sample-related factors”).

**Discussion**

**Line 245: The sentence "The CLIMOS...." is unnecessary.**

Answer: The sentence was deleted.

**Line 295: Diagnostic validity includes sensitivity (detecting minimum leishmanial DNA concentration) and specificity (detecting Leishmanial DNA only). Precision (triplicate runs giving close Cq results). This could be discussed to add more depth to the discussion.**

Answer: The authors do not clearly understand this point raised by the reviewer. The study aims at evaluating the qPCR sensitivity, as an indirect indicator of the extraction performance. The evaluation of the specificity is not the goal of the study and the qPCR method has been already extensively validated in the literature. Here, for the evaluation of extraction methods, the triplicate extraction of three different vials has been performed to minimize the possible impact of differences in parasite pipetting during the preparation of parasite suspensions. The error types on the graphs (Fig.3) reflect both the triplicate amplification and the triplicate extraction (9 Cq values). Regarding error types shown in Fig.1 & 2, it is more the reflect of repeatability of amplification, as only one vial of each concentration was analyzed by each center. Usually, the repeatability is very good for high concentration and fluctuates for low concentrations, due to Poisson’s law. This was added in the discussion.

**Discuss why Cq for L. major is higher than L. infantum.**

Answer: As recommended by the reviewer, we already answered this comment and add sentences in the discussion.

**Line 297: Any explanation why EZI kit performed better than the others?**

Answer: It is difficult to answer precisely, as all these kits are protected by a license. Maybe the EZI kit performed better than the others due to its optimized efficiency in key areas of DNA extraction. For example, it may provide superior cell lysis, resulting in more effective release of DNA, along with better removal of contaminants like proteins and inhibitors.

**Line 301: CLIMOS has been acknowledged for funding, which is enough.**

Answer: The mention of CLIMOS project was deleted.

**Fig 2: High Cq variability between centers ranging from 23 (center 8) to 43 (center 8). Explain.**

Answer: We assume that the reviewer misspelled center 7 (Ct=43) instead of center 8. Variations between centers are quite normal, as they involve different experimentally infected sand flies. During experimental infections, it is not possible to standardize infection rates between individuals. In fact, each individual responds differently to infection, depending on factors such as the size of the bloodmeal or the age of the female. This point was explained in the Discussion (lines 296-300): “This difference underscores the heterogeneity in host-pathogen interactions at the individual level, even under standardized infection conditions. Indeed, this variability could be the result of fluctuations which are expected and deemed normal within the context of experimental infection, influenced by factors such as the size of the blood meal and the age of the sand fly, among others (28).”

**Fig 1 and 2 are reflecting the efficiency of the centers in the detecting the leishmanial DNA as a function of Cq value depending on the setup they are using such as the extraction kit, extraction machine, and thermal cycler. This has not been discussed although the title of the manuscript is 'evaluating the nine center...."**

Answer: We thank the reviewer for his comment. However, the discussion section already mentioned that the Cq values could depend on the choice of extraction kit, the specific methodologies and equipment employed, and setup of positive threshold (lines 274-275, lines 286-288).

**Reviewer #3:**

**=====================**

**The paper entails sending a panel of equal Leishmania parasite containing samples to different Leishmania reference labs in Europe to compare extraction techniques effect on ability to detect Leishmania DNA while controlling for qPCR reagents and amplification conditions. The results demonstrate that all centers are able to detect pathogen in the surrogate surveillance samples but also showed the different nucleic acid isolation kits and techniques do introduce certain levels of variation in the Cq of the results. This is important work to do periodically to ensure detection mechanisms are still comparable and sufficient, offer data to support standardization of DNA isolation kits used for epidemiological surveillance purposes and identify centers that need to improve techniques. The study is well designed and straightforwardly presented. Nice work and useful, practical information for the field.**

**Minor Revisions:**

**Line 41, 134, 140: Leishmania infantum and major are different species, not strains. Please replace.**

Answer: strains was replace by species.

**Line 49, ‘sand fly’ should be ‘sand flies’**

Answer: “sand fly” was replace by “sand flies”

**Line 61, infected is misspelled.**

Answer: The word “infected” had been corrected.

**Line 144, shoud read “Ready to use,”**

Answer: The sentence was corrected.

**Line 153 and 194, grinded should instead by ‘ground’**

Answer: grinded was replaced by ground.

**Table 2, title or legend should indicate what is being compared (Cq value) in the table.**

Answer: Legends of Title 2 and 3 were corrected according to reviewer comment.

**Table 2, to make it easier to read and find the comparisons of interest you could split the Kit Compared column into two, and merge the cells for each of kit 1. Example:**

| EZ1 DSP Virus® Kit | EZ1 DSP Virus® Kit |
| --- | --- |
|  | EZ1 DSP Virus® Kit |
|  | EZ1 DSP Virus® Kit |
|  | EZ1 DSP Virus® Kit |
|  | EZ1 DSP Virus® Kit |
|  | EZ1 DSP Virus® Kit |
| RSC Blood DNA® v | EZ1 DSP Virus® Kit |
|  | EZ1 DSP Virus® Kit |
|  | EZ1 DSP Virus® Kit |
|  | EZ1 DSP Virus® Kit |
|  | EZ1 DSP Virus® Kit |
|  | EZ1 DSP Virus® Kit |
| QIAamp® viral RNA mini kit v | EZ1 DSP Virus® Kit |
|  | EZ1 DSP Virus® Kit |
|  | EZ1 DSP Virus® Kit |
|  | EZ1 DSP Virus® Kit |
|  | EZ1 DSP Virus® Kit |
|  | EZ1 DSP Virus® Kit |

Answer: Tables 2&3 were slightly modified for better clarity.

**Line 241, should read in the presence of “sand fly DNA”**

Answer: The sentence was corrected.

**Line 258, should read “such as inaccurate”**

Answer: The sentence was corrected.

**Major Revisions:**

**Did any centers have amplification/obtain a Cq value on the DNA from the uninfected sand fly DNA? In my experience, unfed or male sand flies sometimes amplify with a late Cq value using kDNA primers. Reporting the background amplification Cq obtained on the negative flies with the different extraction kits could be valuable information and determine if the higher Cq obtained by Center 1 in the Fig 4 is actually above background. It could be added in the same graph. If no amplification of negative sand fly DNA was observed by any centers, that is also worth mentioning in the results section.**

Answer: Amplification was never observed from uninfected samples. False signals of amplification for negative sand flies can sometimes result from unstable annealing of the probe and can be easily detected by visualizing the amplification curve in linear mode: a line is observed instead of an asymptote curve.

**General comments:**

**=====================**

**Figure Files:**

**While revising your submission, please upload your figure files to the Preflight Analysis and Conversion Engine (PACE) digital diagnostic tool, https://pacev2.apexcovantage.com. PACE helps ensure that figures meet PLOS requirements. To use PACE, you must first register as a user. Then, login and navigate to the UPLOAD tab, where you will find detailed instructions on how to use the tool. If you encounter any issues or have any questions when using PACE, please email us at figures@plos.org.**

Answer: Figures were upload in PACE and named and corrected according to PLOS requirements.

**Data Requirements:**

**Please note that, as a condition of publication, PLOS' data policy requires that you make available all data used to draw the conclusions outlined in your manuscript. Data must be deposited in an appropriate repository, included within the body of the manuscript, or uploaded as supporting information. This includes all numerical values that were used to generate graphs, histograms etc.. For an example see here:** [**http://www.plosbiology.org/article/info%3Adoi%2F10.1371%2Fjournal.pbio.1001908#s5**](http://www.plosbiology.org/article/info%3Adoi%2F10.1371%2Fjournal.pbio.1001908#s5)**.**

Answer: All the data were provided as supplementary data.

**Reproducibility:**

**To enhance the reproducibility of your results, we recommend that you deposit your laboratory protocols in protocols.io, where a protocol can be assigned its own identifier (DOI) such that it can be cited independently in the future. Additionally, PLOS ONE offers an option to publish peer-reviewed clinical study protocols. Read more information on sharing protocols at** [**https://plos.org/protocols?utm_medium=editorial-email&utm_source=authorletters&utm_campaign=protocols**](https://plos.org/protocols?utm_medium=editorial-email&utm_source=authorletters&utm_campaign=protocols)

Answer: The detailed laboratory protocol will be available after publication on the website of CLIMOS project.
